# Targeting IL-6 by engineered *Lactococcus lactis* via surface-displayed affibody

**DOI:** 10.1186/s12934-022-01873-7

**Published:** 2022-07-16

**Authors:** Abida Zahirović, Aleš Berlec

**Affiliations:** 1 Department of Biotechnology, Jožef Stefan Institute, Ljubljana, Slovenia; 2 Faculty of Pharmacy, University of Ljubljana, Ljubljana, Slovenia

**Keywords:** Inflammatory bowel disease, IL-6, Microbiota, *Lactococcus lactis*, Delivery system

## Abstract

**Background:**

Dysregulated production of interleukin (IL)-6 is implicated in the pathology of inflammatory bowel disease (IBD). Neutralization of IL-6 in the gut by safe probiotic bacteria may help alleviate intestinal inflammation. Here, we developed *Lactococcus lactis* with potent and selective IL-6 binding activity by displaying IL-6-specific affibody on its surface.

**Results:**

Anti-IL-6 affibody (designated as ZIL) was expressed in fusion with lactococcal secretion peptide Usp45 and anchoring protein AcmA. A high amount of ZIL fusion protein was detected on bacterial surface, and its functionality was validated by confocal microscopy and flow cytometry. Removal of IL-6 from the surrounding medium by the engineered *L. lactis* was evaluated using enzyme-linked immunosorbent assay. ZIL-displaying *L. lactis* sequestered recombinant human IL-6 from the solution in a concentration-dependent manner by up to 99% and showed no binding to other pro-inflammatory cytokines, thus proving to be highly specific for IL-6. The removal was equally efficient across different IL-6 concentrations (150–1200 pg/mL) that were found to be clinically relevant in IBD patients. The ability of engineered bacteria to capture IL-6 from cell culture supernatant was assessed using immunostimulated human monocytic cell lines (THP-1 and U-937) differentiated into macrophage-like cells. ZIL-displaying *L. lactis* reduced the content of IL-6 in the supernatants of both cell lines in a concentration-dependent manner by up to 94%. Dose response analysis showed that bacterial cell concentrations of 10^7^ and 10^9^ CFU/mL (colony forming units per mL) were required for half-maximal removal of recombinant and macrophage-derived IL-6, respectively.

**Conclusion:**

The ability of ZIL-displaying *L. lactis* to bind pathological concentrations of IL-6 at common bacterial doses suggests physiological significance.

**Supplementary Information:**

The online version contains supplementary material available at 10.1186/s12934-022-01873-7.

## Background

Environmental factors, including chemical, mechanical, or pathogen-derived stimuli, can damage the intestinal mucosal barrier and set off inflammation in genetically susceptible individuals, leading to the development of inflammatory bowel disease (IBD). Crohn’s disease and ulcerative colitis are two forms of IBD characterized by overactive immune cells and excessive cytokine response in the intestinal mucosa. Cytokines are central mediators of inflammatory processes during both the active and chronic phases of IBD. They not only promote intestinal inflammation but can also cause extra-intestinal symptoms (such as arthritis). In patients with longstanding IBD, the recurrent mucosal inflammation can induce malignant transformation of epithelial cells and increase the risk of colorectal cancer [1]. Cytokines that drive the development of IBD include tumor necrosis factor (TNF), interleukin (IL)-6, IL-8, IL-17, IL-11, IL-18 and IL-23 [2].

Neutralization of cytokines has become an established treatment strategy for IBD. Systemic administration of anti-TNF antibodies is now routinely used in the clinic. It can be highly effective, but many problems remain, including serious systemic side effects, high treatment costs and lack of efficacy in certain groups of patients. Studies have shown, that anti-TNF therapy is ineffective in up to 50% of patients, more than half of whom become unresponsive over time [3]. These drawbacks warrant the development of alternative therapeutics for patients who are resistant to anti-TNF therapy. Therefore, in addition to TNF, other cytokines involved in the pathogenesis of IBD have been considered as targets [4, 5]. Among these, IL-6 is of great therapeutic interest. IL-6 has been shown to prevent apoptosis of mucosal T cells in IBD by inducing the anti-apoptotic genes Bcl-xl and Bcl-2 [1]. The ensuing T cell expansion perpetuates chronic intestinal inflammation. Augmented local production and increased serum levels of IL-6 have been found in IBD patients [6, 7]. Moreover, recent studies have demonstrated a clear association between IL-6 serum levels and disease severity/relapse [8].

Biologics directed against IL-6 have shown promise in clinical trials. A monoclonal antibody targeting the IL-6 receptor induced a significant clinical improvement in patients with active Crohn’s disease [9]. Furthermore, in a recent phase 2 clinical trial, administration of the antibody against IL-6 resulted in high remission rates in patients with Crohn’s disease, who had previously failed to respond to anti-TNF therapy [10]. Monoclonal antibodies that interfere with IL-17 signaling axis [4] and IFN-γ [5] have also been studied, but have been less successful in clinical setting. On the other hand, monoclonal antibody that blocks IL-23 and IL-12 has been registered for Crohn’s disease and ulcerative colitis.

Apart from using monoclonal antibodies, many cytokines can be neutralized by high-affinity non-immunoglobulin binders [11, 12], which are developed through a biopanning of complex combinatorial libraries of protein variants [13]. Unlike immunoglobulins, these proteins are small, generally do not contain disulfide bonds, and have simple folding [14]. Examples include designed ankyrin repeat proteins, affibodies, fynomers, affitins, and adnectins [13]. Recently, Yu et al*.* developed IL-6-binding affibody via selection from a phage-displayed library [15]. Several other non-immunoglobulin antagonists of the IL-6 signaling are in development, such as repebodies [16], aptamers [17] and peptides [18].

Because cytokines are involved in the host defence response to pathogens and damaged cells, as well as in pathology, systemic administration of anti-cytokine agents can cause side effects, including severe opportunistic infections and malignancies [19]. This can be reduced or avoided by local administration of cytokine inhibitors to the site of inflammation in GIT. For that purpose, oral protein delivery systems that can protect biologics from degradation in the stomach and duodenum are being developed [20]. Bacteria represent such an option that can be used as a microbial cell factory and, at the same time, as an oral delivery system for cytokine-binding proteins. This eliminates the need for expensive production and purification of recombinant proteins in eukaryotic cells.

Food-grade lactic acid bacteria (LAB), such as *Lactococcus lactis* (*L. lactis*), represent a suitable expression host for the development of oral biologics [21]. This species is relatively resistant to gastric acid and bile salts, thrives in the intestinal environment, but does not colonize the gastrointestinal tract (GIT) and therefore has a low potential to negatively affect gut microbiota [22]. *L. lactis* has been used as a vehicle for the delivery of various functional proteins to the intestinal mucosa [14, 23–26]. Delivery of cytokine-binding proteins into the gut by viable *L. lactis* has been shown in a mouse model of ulcerative colitis [23]. An important advantage of using *L. lactis* for IBD treatment relates to its immunostimulatory [27, 28] and probiotic properties [29] since microbial imbalance (dysbiosis) plays a crucial role in the pathology of the disease. Probiotic administration has been shown to correct dysbiosis in IBD by preventing the expansion of opportunistic pathogens, reducing gut inflammation, and restoring immunologic and metabolic homeostasis [30].

Harnessing these beneficial probiotic properties, we set out to develop *L. lactis* as a carrier of the anti-IL-6 affibody that will be able to decrease the content of free IL-6 in the intestine and thus block its detrimental effects in IBD. The surface display of cytokine protein binders may protect them from harsh conditions in the intestinal environment and enable the removal of IL-6 along with the bacteria after they have passed through the gut. For therapeutic application in IBD, we previously developed *L. lactis* displaying on its surface the binders of TNF [31], IL-17A [11], IL-23 [12] or a combination of these [32]. *L. lactis* displaying TNF-specific affibody was tested in vivo, in a mouse model of colitis [33]. In the present study, we displayed IL-6 specific affibody on the surface of *L. lactis* and characterized in detail the capacity of engineered bacteria to remove IL-6 from the surrounding medium in vitro.

## Results

### Construction of plasmid for display of IL-6-binding affibody ZIL on *L. lactis* surface

**Fig. 1:**
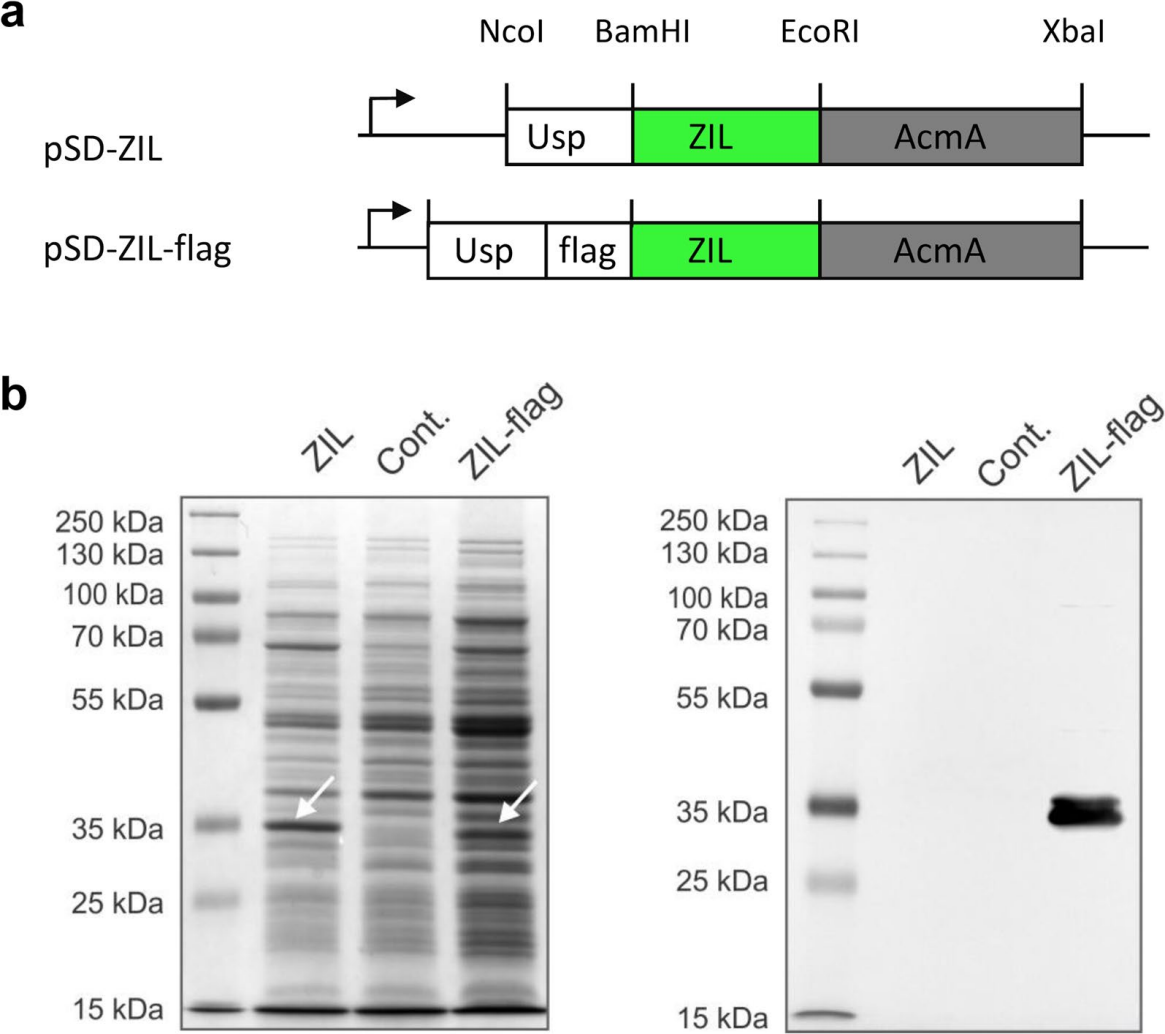
IL-6 binding affibody ZIL is expressed in *L. lactis*. **a** Gene constructs for expression of IL-6 binding affibody ZIL on the surface of *L. lactis*. USP, gene encoding Usp45 secretion signal (84 bp). ZIL, gene encoding IL-6-binding affibody (174 bp). Flag, epitope tag sequence. AcmA, gene encoding C-terminal domain of AcmA anchoring protein (642 bp). The arrow represents the nisin-inducible promoter. **b** Coomassie brilliant blue-stained SDS-PAGE gel (left) and Western blot analysis (right) of whole lysates of *L. lactis* harbouring plasmids pSD-ZIL or pSD-ZIL-flag. Cont., *L. lactis* containing empty plasmid pNZ8148. Bands representing untagged or flag-tagged ZIL fusion protein are indicated with arrows

The gene encoding the IL-6-binding protein ZIL was codon-optimized for *L. lactis*, synthesized and cloned into a lactococcal plasmid for surface display as described in Materials and methods. A flag tag sequence (DYKDDDDK) was attached to the N-terminus to facilitate detection by antibodies. The binder was fused to the Usp45 signal peptide and the AcmA anchor to achieve its secretion into the growth medium and subsequent binding onto the bacterial surface (Fig. 1a). The band with an apparent molecular weight of ∼35 kDa was detected in the whole lysate of host cells by Coomassie Blue staining and Western blot analysis, whereas no signals were present in the cell lysate of control cells harbouring the empty plasmid pNZ8148 (Fig. 1b). The size of the band corresponds to the predicted molecular mass of the ZIL fusion protein (ZIL ∼7 kDa and AcmA ∼25 kDa), with no traces of protein degradation. The double band represents unprocessed protein (with signal peptide) and secreted, mature protein (without signal peptide). To confirm that the nisin promoter was activated, we compared protein expression in induced and uninduced bacterial cultures. The bands representing ZIL fusion protein were present in the whole cell lysate of induced cultures, while they were not present in uninduced cultures (Additional file 1: Fig. S1).

### IL-6-binding affibody ZIL is displayed on the surface of *L. lactis*

**Fig. 2:**
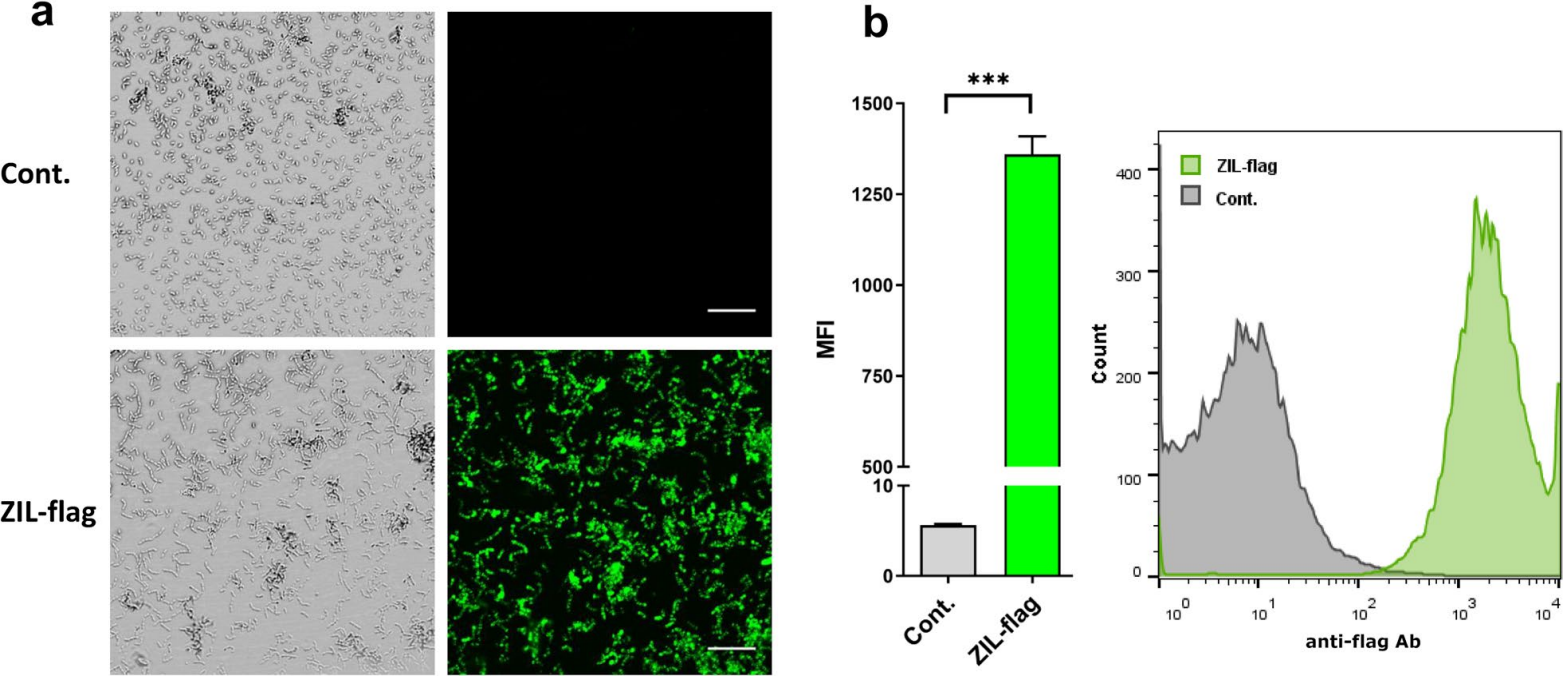
IL-6-binding affibody ZIL is displayed on *L. lactis* surface. **a** Representative confocal immunofluorescence microscopy images visualizing ZIL-flag at the bacterial surface and **b** flow cytometry analysis showing a large increase in mean fluorescence intensity and a shift of the population of ZIL-flag-expressing *L. lactis* compared to control bacterial cells. ZIL-flag, *L. lactis* harbouring pSD-ZIL-flag plasmid. Cont., *L. lactis* harbouring empty plasmid pNZ8148. Engineered bacteria were incubated with an anti-flag antibody and then probed with Alexa Fluor 488 or Alexa Fluor 555-conjugated secondary antibody. The results are expressed as mean ± standard deviation (SD) of three individual measurements. ***, P < 0.001 (unpaired t-test). **a**: Bright-field images (left) and the corresponding fluorescence images (right). Bar scale 20 µm. **b**: MFI, mean fluorescence intensity

To determine whether the expressed ZIL fusion protein is displayed on the bacterial surface, intact bacteria harbouring pSD-ZIL-flag plasmid were stained with anti-flag antibodies and evaluated by confocal microscopy and flow cytometry. Microscopy showed strong fluorescence staining of the ZIL-flag-expressing *L. lactis*, demonstrating the presence of ZIL on the bacterial cell surface, whereas no fluorescence signal was observed in the control *L. lactis* harbouring the empty plasmid (Fig. 2a). This was substantiated by flow cytometry, where we observed a large increase in mean fluorescence intensity (approximately 300-fold higher signal) and a distinct shift in the population of ZIL-flag-expressing *L. lactis* cells compared to the control bacteria (Fig. 2b).

### Surface-displayed IL-6-binding affibody ZIL is functional

**Fig. 3:**
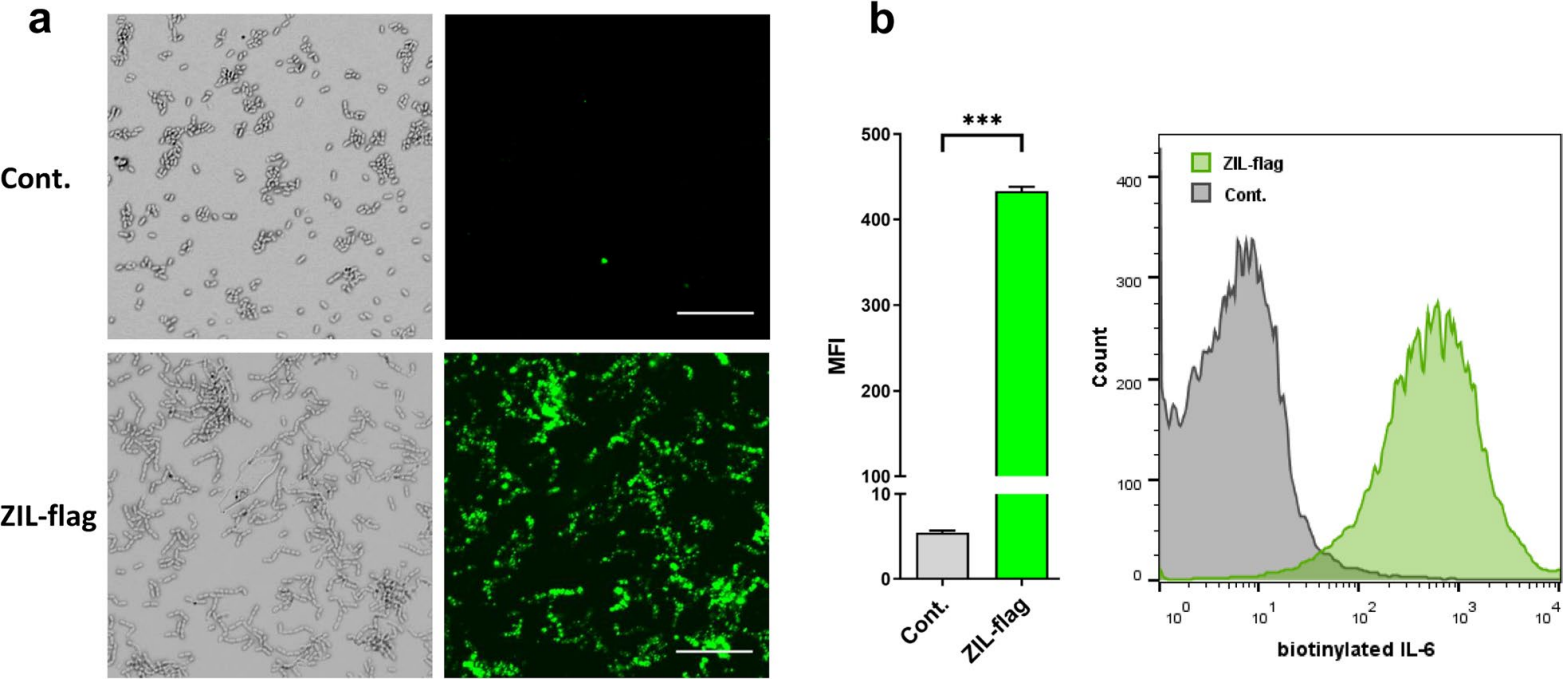
Surface-displayed IL-6-binding affibody ZIL is functional. Representative confocal immunofluorescence microscopy images (**a**) and flow cytometric analysis (**b**) showing binding of ZIL-flag-displaying *L. lactis* to human biotin-conjugated IL-6. ZIL-flag., *L. lactis* cells containing pSD-ZIL-flag plasmid. Cont., *L. lactis* control cells containing empty plasmid pNZ8148. Control or ZIL-flag-displaying *L. lactis* cells were incubated with IL-6-biotin and detected with an anti-biotin antibody, followed by a secondary antibody conjugated to Alexa Fluor 488. The results are presented as mean ± standard deviation (SD) of three individual measurements. ***, P < 0.001 (unpaired t-test). **a**: Bright-field images (left) and the corresponding fluorescence images (right). Bar scale 20 µm. **b**: MFI, mean fluorescence intensity

The functionality of surface-displayed IL-6-binding affibody was assessed by exposing ZIL-flag-displaying *L. lactis* to human biotinylated IL-6 and analyzing its binding to the bacteria by confocal microscopy and flow cytometry. ZIL affibody was isolated by Yu et al. [15] via biopanning on biotinylated human IL-6, which was therefore used as a molecular probe to test ZIL functionality. Both confocal microscopy and flow cytometry showed that ZIL-flag-displaying *L. lactis* can bind biotinylated IL-6, whereas the control *L. lactis* showed no binding (Fig. 3a and b). ZIL-flag-displaying *L. lactis* cells incubated in the presence of biotinylated IL-6 exhibited bright fluorescence as revealed by confocal microscopy at the single-cell level (Fig. 3a). Consistent with the result of the fluorescence imaging, an increase in the mean fluorescence intensity of ZIL-flag-displaying *L. lactis* cells was observed by flow cytometry with a clear shift in the emission peak (Fig. 3b). The mean fluorescence intensity of ZIL-flag-displaying *L. lactis* cells was 80-fold higher than that of empty plasmid control cells (Fig. 3b).

### ZIL-displaying *L*. *lactis* specifically removes various amounts of recombinant IL-6 from the solution in a concentration-dependent manner

**Fig. 4:**
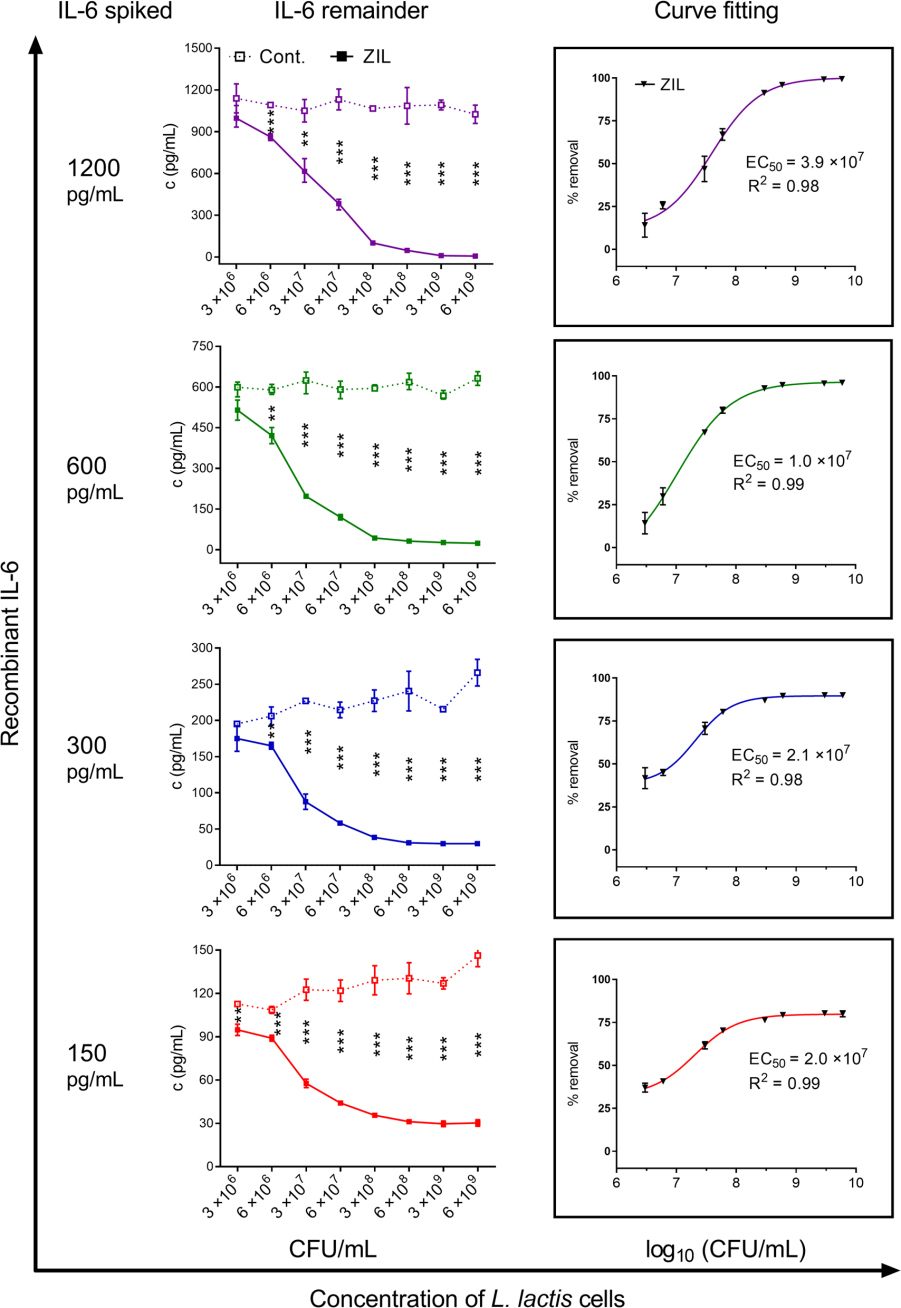
ZIL-displaying *L. lactis* removes various amounts of recombinant IL-6 from the solution in a concentration-dependent manner. ELISA-determined concentrations of recombinant IL-6 that remained in the solution following incubation with ZIL-displaying *L. lactis* (3 × 10^6^–6 × 10^9^ CFU/mL) across four concentrations of recombinant IL-6 (150, 300, 600 and 1200 pg/mL) that were spiked into the PBS buffer (left). ZIL., *L. lactis* cells containing pSD-ZIL plasmid. Cont., *L. lactis* control cells containing empty plasmid pNZ8148. Dose response curves for calculating the concentration of ZIL-displaying bacterial cells necessary to remove a 50% of recombinant IL-6 from the solution (half-maximal effective concentration, EC_50_) determined by curve fitting with four parameters logistic (4 PL) regression model in GraphPad Prism (right). The results are expressed as mean ± standard deviation (SD) of three individual measurements. **, P ≤ 0.006; ***, P < 0.001 (unpaired t-test)

A dose response analysis was performed to assess the capacity of ZIL-displaying *L. lactis* to remove IL-6 from solution at various concentrations of bacterial cells ranging from 3 × 10^6^ to 6 × 10^9^ CFU/mL. Recombinant human IL-6 was spiked into the PBS buffer to the final concentration of 150, 300, 600 and 1200 pg/mL and the amount that remained after the incubation with ZIL-displaying *L. lactis* was determined by ELISA. An increase in bacterial cell concentration resulted in increased removal of IL-6 from the solution. The level of removal correlated with the concentration of bacterial cells across all IL-6 concentrations tested; it was statistically significant at 10^6^ CFU/mL and above (Fig. 4). To determine the half-maximal effective concentration (EC_50_), the percentage of IL-6 removal was plotted against log-transformed bacterial concentrations and fitted to a four-parameter sigmoidal curve (4PL regression model). EC_50_ is defined as the concentration at which 50% removal is achieved. The estimated EC_50_ of ZIL-displaying *L. lactis* for recombinant IL-6 was 10^7^ CFU/mL. To confirm that IL-6 binds to ZIL moiety of the fusion protein and not to its other components (i.e. Usp-flag and AcmA), we tested IL-6 binding to control *L. lactis* cells that display Usp-flag and AcmA in fusion with nonrelevant binders (IL-8-binding evasin and HER2-binding affibody). The control strains exhibited no binding to human IL-6 (Additional file 1: Fig. S2), which confirms that neither Usp-flag nor AcmA binds IL-6.

**Fig. 5:**
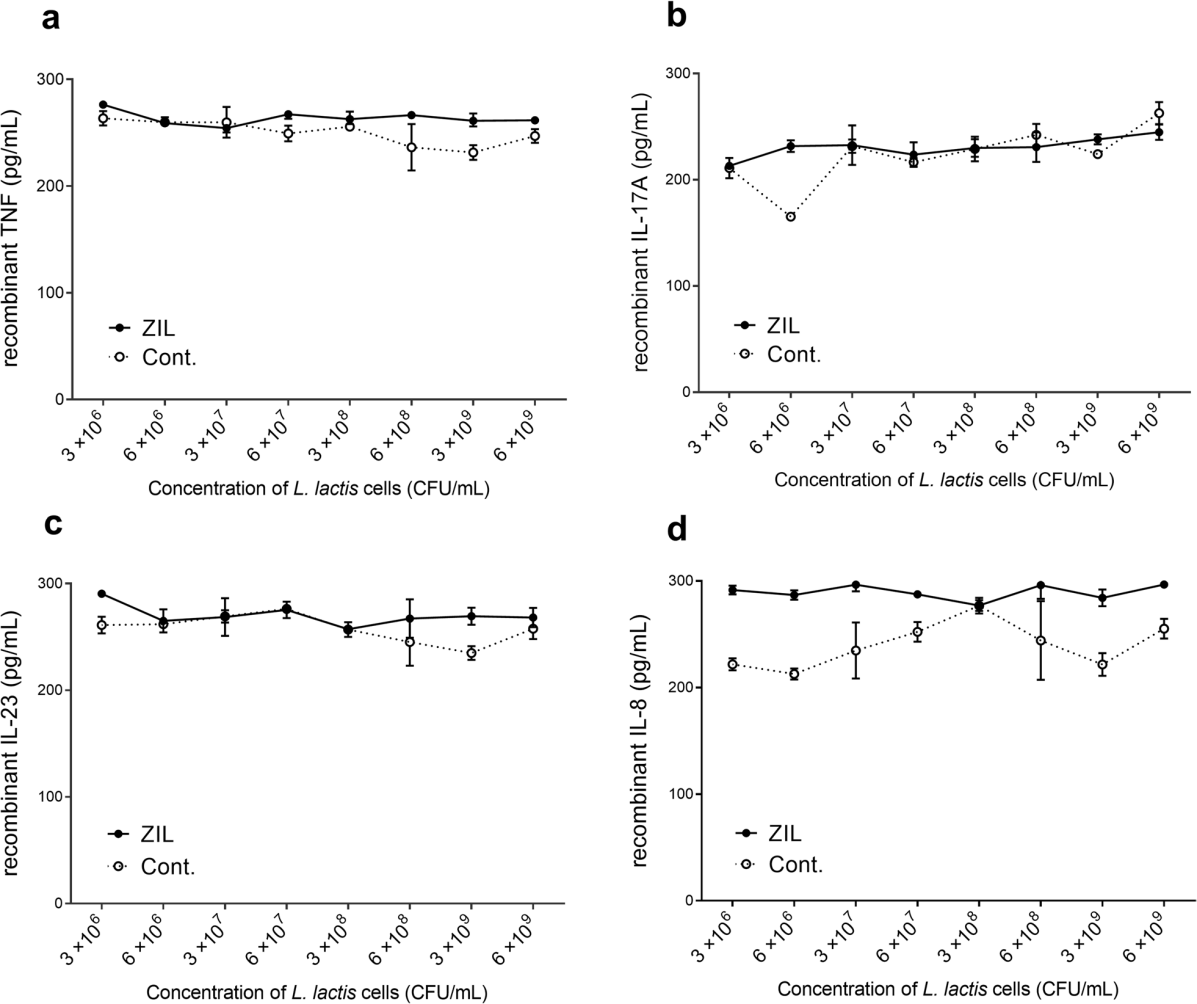
ZIL-displaying *L. lactis* does not bind TNF, IL-17, IL-23 or IL-8. ELISA-determined concentration of recombinant TNF (**a**), IL-17 (**b**), IL-23 (**c**), IL-8 (**d**) that remained in the solution following their incubation with increasing concentrations of ZIL-displaying *L. lactis* (ZIL). Cont., *L. lactis* control cells containing empty plasmid pNZ8148. The experiments are performed in triplicate. Data are means ± standard deviation (SD)

To test their specificity, ZIL-displaying *L. lactis* were exposed to pro-inflammatory cytokines TNF, IL-17, IL-23, and IL-8 that also drive pathology of IBD besides IL-6. ZIL-displaying *L. lactis* showed no binding to these cytokines, demonstrating that they are specific only for IL-6 (Fig. 5).

**Fig. 6:**
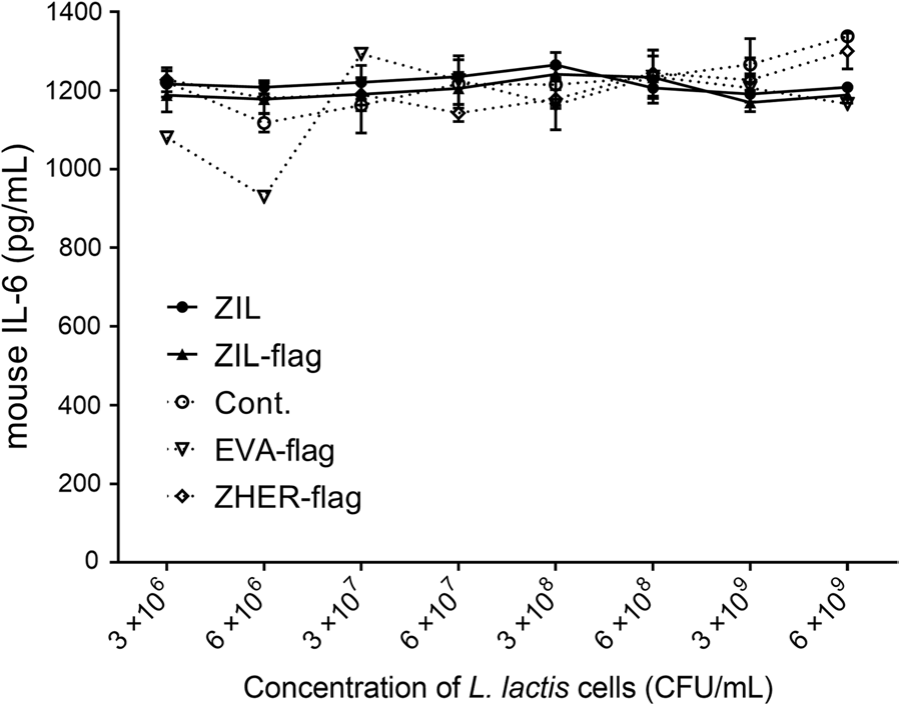
ZIL-displaying *L. lactis* does not cross-react with mouse IL-6. ELISA-determined concentration of recombinant mouse IL-6 that remained in the solution following incubation with increasing concentrations of ZIL-displaying *L. lactis* (ZIL) and ZIL-flag-displaying *L. lactis* (ZIL-flag). Cont., *L. lactis* control cells containing empty plasmid pNZ8148. EVA-flag, *L. lactis* control cells containing pSD-EVA-flag plasmid. ZHER-flag, *L. lactis* control cells containing pSD-ZHER-flag plasmid. The experiment is performed in triplicate. Data are means ± standard deviation (SD)

Further, we tested the species specificity of recombinant bacteria and found that ZIL-displaying *L. lactis* does not cross-react with mouse IL-6 (Fig. 6).

### ZIL-displaying *L. lactis* removes IL-6 secreted by differentiated THP-1 and differentiated U-937 cells in proportion to the concentration of bacterial cells

ZIL-displaying *L. lactis* was further assessed for the ability to remove IL-6 secreted by immune cells implicated in IBD pathogenesis. Many cell types have been shown to produce IL-6 including monocytes, macrophages, T lymphocytes, endothelial cells, fibroblasts, and tumour cells. In the inflamed mucosa of IBD patients, macrophages are responsible for the bulk of IL-6 activity [34]. For assaying the ability of ZIL-displaying *L. lactis* to remove macrophage-derived IL-6, monocytic THP-1 cells and U-937 cells were differentiated into macrophage-like cells by incubation with PMA. The differentiation was verified by phase-contrast microscopy, which showed that the cells underwent a morphological change upon PMA treatment. While undifferentiated cells were round, grew in suspension and gathered into clusters, PMA-induced cells became adherent and spindle-shaped with cellular extensions and more conspicuous granules (Additional file 1: Fig. S3a). To evoke IL-6 secretion, differentiated cells were primed with LPS. The supernatants of the stimulated cells were collected at different time points and the kinetics of IL-6 secretion was analysed. As shown in Additional file 1: Fig. S3b, the production of IL-6 increased in a time-dependent manner after LPS treatment in both cell lines. Differentiated THP-1 cells responded immediately to LPS stimulation; the concentration of IL-6 began to rise 2 h after initial exposure and gradually increased during 24 h of culture, reaching 1572 pg/mL. In differentiated U-937 cells, secretion kinetics were essentially the same, except that the levels of released IL-6 were considerably higher, reaching up to 4435 pg/mL.

**Fig. 7:**
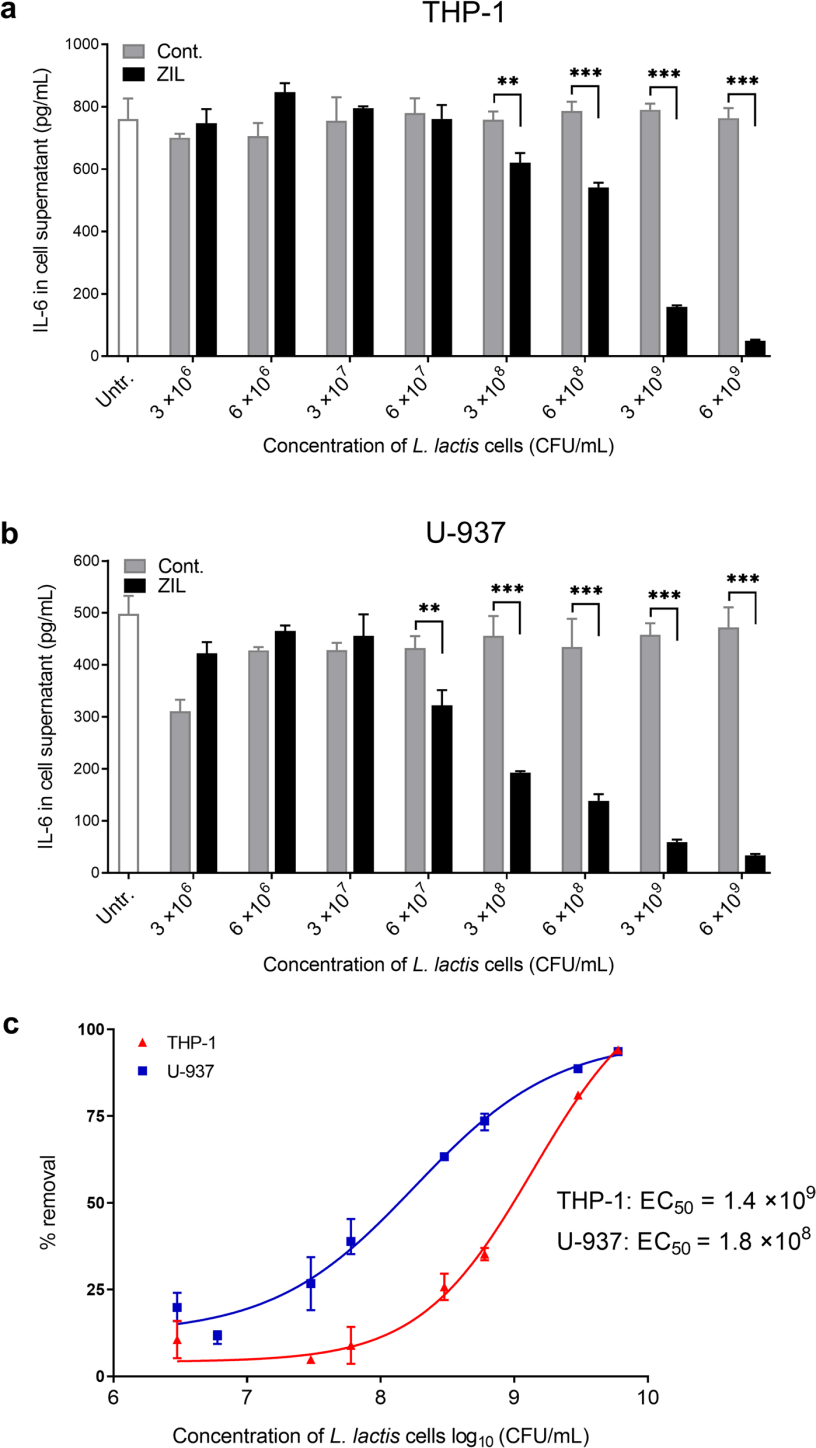
ZIL-displaying *L. lactis* removes IL-6 secreted by differentiated THP-1 and differentiated U-937 cells in proportion to the concentration of bacterial cells. ELISA-determined concentrations of IL-6 that remained in the supernatants of LPS-induced differentiated THP-1 cells (**a**) and differentiated U-937 cells (**b**) following incubation with ZIL-displaying *L. lactis* (ZIL). Cont., *L. lactis* control cells containing empty plasmid pNZ8148. Untr., untreated supernatants. Dose response curves for calculating the concentration of ZIL-displaying *L. lactis* cells necessary for removal of a 50% of IL-6 from the cell culture supernatants (EC_50,_ half-maximal effective concentration) was determined by curve fitting using four parameters logistic (4 PL) regression model in GraphPad Prism (**c**). The results are expressed as mean ± standard deviation (SD) of experiments performed in triplicate. **, P ≤ 0.007; ***, P < 0.001 (unpaired t-test)

To assess the removal of macrophage-derived IL-6 by ZIL-displaying *L. lactis,* supernatants collected from differentiated cells were incubated with increasing concentrations of engineered bacteria (from 3 × 10^6^ to 6 × 10^9^ CFU/mL) and the proportion of the removed IL-6 was determined by ELISA. After incubation with ZIL-displaying *L. lactis*, the amount of IL-6 was considerably reduced in the supernatants of both cell lines, whereas the concentration of IL-6 remained essentially unchanged in the supernatants incubated with empty plasmid control *L. lactis* (Fig. 7a and b). IL-6 removal correlated with the concentration of bacterial cells, it was statistically significant at 6 × 10^7^ CFU/mL and above. Dose response relationship was analysed by regressing the percentage of IL-6 removal against log-transformed bacterial concentration. The results showed that the removal was more efficient from the supernatant of differentiated U-937 cells, where 10^8^ CFU/mL ZIL-displaying *L. lactis* was required to capture 50% of secreted IL-6, whereas ten times more cells were required to remove a similar amount of IL-6 secreted by differentiated THP-1 cells (Fig. 7c). At the highest bacterial concentration tested (6 × 10^9^ CFU/mL), ZIL-displaying *L. lactis* removed above 90% of IL-6 from the supernatant of both cell lines. Notably, even at a relatively low concentration (3 × 10^8^ CFU/mL), ZIL-displaying *L. lactis* reduced IL-6 levels in the cell supernatant by up to 61%.

## Discussion

Protein binders of pro-inflammatory cytokines delivered locally into the gut by engineered live bacteria have been shown to reduce inflammation in the mouse models of IBD [23]. Non-pathogenic lactic acid bacteria provide a suitable vehicle for oral protein delivery. Recently, *L. lactis*, a model lactic acid bacterium, has been used as an expression host and a mucosal delivery vector for binders of TNF [33], IL-17A [11], or IL-23 [12]. IL-6 is a key modulator of immune responses in IBD and therefore holds promise as a target for novel therapeutic strategies. Here, we displayed IL-6-binding affibody on the surface of *L. lactis* and demonstrated a high degree of IL-6 removal by the engineered bacteria in vitro. Developed *L. lactis* with strong IL-6-binding ability is suitable for further development as an alternative IBD treatment, which combines local neutralization of IL-6 with beneficial effects of oral probiotics.

Anti-IL-6 affibody used in this study (herein referred to as ZIL) was selected as a high-affinity IL-6 binder from a complex phage-displayed library [15]. Affibodies are 58-residue derivatives of the Z domain from staphylococcal protein A containing three-helix bundles with 13 sites on helices 1 and 2 commonly randomized for binding. For expression and display of affibody ZIL on *L. lactis*, the expression cassette consisted of Usp45 secretion signal [35], IL-6-binding domain [15], and AcmA protein anchor [36]. ZIL fusion protein was detected in the lysate of engineered bacteria by both Coomassie blue staining and Western blot analysis.

Surface accessibility of the expressed ZIL fusion protein was confirmed by fluorescence microscopy and flow cytometry. The extent of surface display depends on the properties of the passenger protein including protein yield, molecular weight, physico-chemical properties, and susceptibility to degradation. It is also influenced by the translocation efficiency and secretory protease activity of the bacterial host. A low level of surface display is usually caused by inefficient protein translocation across cell membranes or unfavourable spatial orientation of binder at the bacterial surface [37]. Here, a high level of ZIL surface display was observed and its functionality was validated by demonstrating the ability of ZIL-displaying *L. lactis* to bind biotinylated IL-6. Compared with other small non-immunoglobulin binders, a favourable surface display of affibody scaffold on *L. lactis* has been observed previously [38]. The great surface accessibility favours protein interaction with its target, provided that stability is not compromised. AcmA-anchored proteins were shown to be stable at 4 °C and after incubation in simulated gastric fluid [31, 32].

The ability of ZIL-displaying *L. lactis* to remove IL-6 from the solution was characterized in detail in relation to the concentration of bacterial cells. The removal was highly specific for IL-6, with no cross-reactivity for other pro-inflammatory cytokines implicated in the pathology of IBD. The proportion of captured recombinant IL-6 correlated with the bacterial concentration, and it was statistically significant even at 10^6^ CFU/mL. This is in accordance with a previous study, where the binding of IL-8 by evasin-displaying *L. lactis* was more efficient at higher cell concentrations [39]. Notably, in comparison to evasin-displaying *L. lactis*, ZIL-displaying *L. lactis* exhibited higher removal efficiency even though ZIL and evasin have similar affinity constant for their target cytokines (Kd ~ 430 pM for EVA and Kd ~ 500 pM for ZIL). This confirms previous findings that, apart from affinity, various other factors influence the interaction of surface-displayed binder with its target molecule including the amount of the protein present on the surface, and accessibility of its active site.

In a cell-based assay, we assessed the ability of ZIL-displaying *L. lactis* to remove IL-6 secreted by immune cells that drive intestinal inflammation. Macrophages are predominant producers of IL-6 during the acute phase of IBD [40]. IL-6, released in the early phase of immune reaction, leads to the recruitment of neutrophils and antigen-presenting cells into inflamed tissues. We established a cell culture model of macrophages by treating human monocytic THP-1 and U-937 cells with PMA. LPS treatment of differentiated cells induced strong and rapid secretion of IL-6. After incubation with ZIL-displaying *L. lactis*, a marked decrease in IL-6 levels in the supernatant of both cell lines was observed in a dose-dependent manner (by up to 94%).

We used different concentrations of IL-6 to test removal efficiency of engineered bacteria because multiple studies have reported a wide concentration range of IL-6 in serum and colon tissue of IBD patients [7]. In the study by Mitsuyama et al., serum levels of IL-6 ranged from 4 to 218 pg/mL in patients with active IBD [6]. The average amount of IL-6 in the intestinal mucosa was 54 pg/mg protein in IBD patients compared to 5 pg/mg protein in healthy individuals [6]. Here, ZIL-displaying *L. lactis* was equally efficient in removing 150, 300, 600, and 1200 pg/mL of recombinant IL-6. Also, it was capable of capturing up to 800 pg/mL of IL-6 secreted by macrophage-like cells. The potency of the engineered bacteria was defined by EC_50_, which represents the concentration at which half-maximal removal occurs. By performing dose response analysis, we found that 10^7^ CFU/mL and 10^9^ CFU/mL were required to remove 50% of recombinant and macrophage-derived IL-6, respectively. The difference in potency of ZIL-displaying *L. lactis* for recombinant and natural IL-6 may be due to glycosylation of cell-derived IL-6 [41]. IL-6 from macrophage-like cells contain multi-branched N- and O-glycans that may sterically hinder its interaction with affibody on the bacterial surface. The effect of IL-6 glycosylation on its interaction with the binder was previously reported for anti-IL-6 aptamer, which showed a one-fourth reduction in potency for glycosylated IL-6 compared to non-glycosylated IL-6 [17]. Interestingly, EC_50_ for THP-1 cell-derived IL-6 differ from EC_50_ for U-937 cell-derived IL-6. This may arise from the cell type-specific glycosylation pattern [42] due to the different source and maturation stages of U937 and THP-1 cells [43]. Overall, the removal of pathological amounts of IL-6 by usual therapeutically effective doses of probiotics in humans (10^7^–10^9^ CFU/mg per day) [44] suggests the therapeutic potential of ZIL-displaying *L. lactis*.

ZIL-displaying *L. lactis* does not cross-react with mouse IL-6. Species specificity of biologics targeted towards human IL-6 has been documented previously for monoclonal antibodies against human IL-6 [45]. Similarly, studies have shown that tocilizumab (monoclonal antibody against human IL-6 receptor) does not block the murine IL-6 receptor [46]. For therapeutic monoclonal antibodies, which have low or no cross-reactivity to orthologous rodent molecules, genetically engineered mice and/or surrogate antibodies have to be used for evaluation of their efficacy and safety in preclinical studies. Humanized mouse models expressing human IL-6 or human IL-6 receptor have been developed [47, 48]. Furthermore, a humanized ligand-receptor system for IL6 in mice has been established by crossing a h*IL6* transgenic mouse with a h*IL6 receptor* transgenic mouse [45]. For studying biological activity of human IL-6 antagonist in vivo, a serum amyloid A mouse model is also used. Serum amyloid A is acute phase protein secreted from liver cells. It is highly increased during inflammation and can be induced by exogenously administered recombinant human IL-6.

The ability of LAB to survive passage through GIT makes them suitable for oral administration and provides a means for local delivery of cytokine blockers to the intestine. Oral administration allows direct interaction of live therapeutic bacteria with the inflamed mucosa and the delivery of the drug in the vicinity of reactive cells. Structural deformities of the mucosal epithelium in IBD patients are expected to facilitate the accumulation of bacteria in the mucus layer and transport of the therapeutic payload to the underlying lamina propria. Indeed, previous studies have shown that anti-TNF nanobodies or trefoil factors delivered locally by *L. lactis* were more effective in reducing signs of colitis than the same proteins administered parenterally, orally or rectally [23, 49]. Furthermore, bacteria can be encapsulated in oral formulations that are designed to enhance drug release in the colon or lower small intestine. Regional targeting of biologics allows for lower dosing and fewer systemic side effects [50].

ZIL-displaying *L. lactis* exhibits superior removal efficiency and great specificity compared to the aforementioned cytokine-targeting *L. lactis* strains [11, 12, 31, 32, 39], thus providing a solid foundation for the future development. In the follow up study, we expressed ZIL in combination with tumor antigen binders to generate bacteria with dual functionality that simultaneously target IL-6 and tumor antigens overexpressed on cancer cells [51]. Such modified strains are intended for selective delivery of cytokine binders into tumors.

Further engineering of the developed ZIL-displaying *L. lactis* bacteria, for example through the display of ligands with the affinity towards other pro-inflammatory cytokines could yield strains with even greater utility and applicability. Namely, the development of effective anti-cytokine therapies has been challenged by cytokine redundancy and compensatory responses. Besides interindividual differences, cytokine function is also influenced by the type and the location of inflammation, the plasticity of immune cells, and changes in the cytokine profiles during the disease. Blockade of a single pro-inflammatory cytokine may therefore be insufficient to provide effective therapy for all patients with IBD [5]. Inhibition of multiple cytokines has been proposed to overcome cytokine redundancy. The expression of two or more anti-cytokine modalities on *L. lactis* can produce an additive or synergistic effect [32]. To facilitate construction of multifunctional bacteria, we have devised a novel engineering tool (modified Bglbrick system) that enables straightforward cloning and efficient expression of multiple proteins in *L. lactis* [52] and can be used for the generation of bacteria that target several cytokines.

The use of environmentally controlled inducible promoters would allow in situ expression of therapeutic proteins in the GIT [53]. Regulatory requirements related to the application of genetically modified organisms can be addressed, for example, by using heterologous surface display. In this approach, recombinant proteins are attached to the surface of wild-type bacteria via peptidoglycan-binding anchor [54].

## Conclusions

Taken together, ZIL-displaying *L. lactis*, developed and characterized herein, exhibited strong and selective removal of human IL-6 in vitro*.* This study demonstrates the feasibility of IL-6 targeting by anti-IL-6 affibody displaying *L. lactis* and establishes its suitability for further studies of modulation of cytokine-driven diseases. IL-6-binding *L. lactis* alone or preferably in combination with the bacteria that bind other pro-inflammatory cytokines may provide an alternative therapeutic strategy for IBD.

## Methods

### Plasmid construction

The bacterial strain, gene, plasmids, and primers used in this study are given in Table 1. The gene encoding the IL-6-binding affibody (*zil*; Table 1) was back-translated from the amino acid sequence ZIL6_13 previously described by Yu et al. [15]. The gene was codon-optimized for *L. lactis* NZ9000 using Gene Designer (ATUM), synthesized as a gBlock (Integrated DNA Technologies, Leuven, Belgium) and amplified by PCR using primers ZIL_F_BamHI and ZIL_R_EcoRI (Integrated DNA Technologies; Table 1) to add BamHI/EcoRI restriction sites. The amplicon was routinely ligated into pJET1.2 (CloneJET PCR Cloning Kit, Thermo Fisher Scientific, Waltham, MA, USA) for cloning purposes. The *zil* gene was then transferred to the lactococcal plasmid for surface display pSDBA3b [31], which is derivative of pNZ8148 plasmid that contains expression cassette for surface display consisting of secretion signal sequence Usp45, gene for protein to be displayed (i.e. b-domain), and gene for anchoring protein AcmA. In pSDBA3b*, b-domain* was replaced with the z*il* via BamHI/EcoRI restriction sites, resulting in pSD-ZIL (Fig. 1a). The flag tag was introduced upstream of *zil* by restricting the z*il* from pSD-ZIL and transferring it to pSD-EVA-flag via BamHI/EcoRI restriction sites, yielding pSD-ZIL-flag (Fig. 1a). Plasmid pSD-EVA containing gene for IL-8-binding evasin (*eva*) was prepared in the study by Škrlec et al. [39] for generation of *L. lactis* that targets IL-8. The flag tag gene was introduced into pSD-EVA as described previously [11]. Briefly, EVA was amplified by PCR with primers Usp1-NcoI/FLAG_Bam_R (Table 1), digested with NcoI and BamHI, and cloned into pSD-EVA that was linearized with the same restriction enzymes. PCR amplification, endonuclease digestion, and DNA ligation were performed according to standard protocols. The resulting plasmids were electroporated into *L. lactis* according to [55], using Gene Pulser II (Bio-Rad, Hercules, CA, USA).

**Table 1:** The bacterial strain, plasmids, primers and gene used in this study

Strain, plasmid, primer or gene	Relevant features or sequence	References
*Strain*		
*L. lactis* subsp. *cremoris* NZ9000	MG1363 *nisRK* Δ*pepN*	NIZO
*Plasmids*		
pNZ8148	pSH71 derivative, P_*nisA*_*,* Cm^r^, nisin-controlled expression	[56, 57]
pSDBA3b	pNZ8148 containing gene fusion of *sp *_*Usp45*_, *b-domain* and *acmA3b*	[31]
pSD-EVA	pNZ8148 containing gene fusion of *sp*_*Usp45*_, *eva* and *acmA3b*	[39]
pSD-EVA-flag	pNZ8148 containing gene fusion of *sp*_*Usp45*_, *flagtag, eva* and *acmA3b*	This study
pSD-ZHER-flag	pNZ8148 containing gene fusion of *sp*_*Usp45*_, *flagtag, zher* and *acmA3b*	[38]
pJET-ZIL	pJET containing a fusion gene of ZIL, tolA protein, and AviTag consensus	This study
pSD-ZIL	pNZ8148 containing gene fusion of *sp*_*Usp45*_, *zil* and *acmA3b*	This study
pSD-ZIL-flag	pNZ8148 containing gene fusion of *sp*_*Usp45*_, *zil-flag* and *acmA3b*	This study
*Primers*		
ZIL_F_BamHI	ATTAGGATCCGTTGACGCTAAATATGCTAAAG	This study
ZIL_R_EcoRI	ATTTGAATTCTTTTGGGGCTTGACTATC	This study
Usp1-NcoI	ATAACCATGGCTAAAAAAAAGATTATCTCAGCTATTTTAATG	[31]
FLAG_Bam_R	GGATCCTTTATCATCGTCGTCTTTATAATCAGCGTAAACACCTGACAACG	[11]
*Gene*		
*zil*	GTTGACGCTAAATATGCTAAAGAGGAACAACGTGCTTGGAGAGAAATTCACTTATTACCTAATCTTACAATCGAACAAATGGCAGCATTCATTTGGAAATTGTTAGATGATCCATCACAATCTTCAGAGTTGTTATCAGAGGCTAAAAAACTTAATGATAGTCAAGCCCCAAAA	This study

*MCS* multiple cloning site; NIZO food research BV (the Netherlands)

### Bacterial growth conditions and protein expression in *L. lactis*

*Lactococcus lactis* NZ9000 was grown in M17 medium (MilliporeSigma, Burlington, MA, USA) containing 0.5% glucose (Fluka AG, Buchs, Switzerland) at 30 °C without aeration. Chloramphenicol (10 µg/mL; Sigma-Aldrich, St. Louis, MO, USA) was added for the selection of plasmid-containing bacteria. Overnight cultures of *L. lactis* harbouring constructed plasmids were diluted 1:50 in M-17 medium supplemented with glucose and chloramphenicol and grown to an optical density at 600 nm (OD_600_) of 0.8 (an exponential growth phase). Protein expression was induced with nisin (25 ng/mL; Fluka) and bacterial cultures were incubated for additional 3 h. After incubation, the bacteria were harvested by centrifugation (5000 × *g*, 10 min, 4 °C) and suspended in the phosphate-buffered saline (PBS) to the appropriate concentration for subsequent analyses. The concentration of *L. lactis* cells was calculated based on OD_600_ with a factor of 1 OD_600_ = 1 × 10^9^ colony forming units (CFU)/mL, determined previously by serial dilutions.

### SDS-PAGE and Western blot analyses

Sodium dodecyl sulfate–polyacrylamide gel electrophoresis (SDS-PAGE) and western blot analysis were performed as described previously [26]. Briefly, following protein expression, bacterial cultures (10 ml) in the stationary growth phase (OD_600_ = 3) were pelleted, resuspended in PBS (400 µl) and stored at − 20 °C. Before gel loading, bacterial samples were sonicated (UPS200S; Hielscher, Teltow, Germany) and denatured by heating to 100 °C in 2 × Laemmli sample buffer with dithiothreitol (Thermo Fisher Scientific). Equal amounts of whole cell bacterial extracts were loaded on 12% gel. The separated proteins were visualized on the gel by staining with Coomassie Brilliant Blue (Polysciences Inc., Warrington, PA, USA) or blotted onto nitrocellulose membrane (GE Healthcare Life Sciences, Marlborough, MA, USA) with the Trans-Blot Turbo System (Bio-Rad, Hercules, USA). Molecular weights were estimated using Page Ruler Plus prestained standards (Thermo Fisher Scientific). To block nonspecific binding, membranes were incubated for 1 h with 5% skim milk in tris-buffered saline (TBS) containing 0.05% Tween-20 (TBST). Subsequently, the membranes were probed overnight at 4 °C with rabbit anti-flag antibody (1:10.000; Proteintech, Rosemont, IL, USA) in blocking buffer. After washing with TBST, membranes were incubated with StarBright IgG Blue 520 fluorescent goat anti-rabbit secondary antibody (1:5.000; Bio-Rad) for 1.5 h at room temperature. ChemiDoc MP Imaging System (Bio-Rad) was used for image acquisition and processing.

### Flow cytometry

Flow cytometry was performed as described previously [58]. Briefly, 8 × 10^7^ CFU of bacterial cells were incubated for 2 h at room temperature with anti-flag antibody (Proteintech) diluted 1:500 in 500 µL TBS or overnight at 4 °C with 1 µg/ml of recombinant human biotin-labeled IL-6 (Immunotools, Friesoythe, Germany) in 500 µL TBS. Anti-flag antibodies were detected with goat anti-rabbit antibody conjugated to Alexa Fluor 488 (Cell Signaling Technology, Danvers, MA, USA). Biotinylated IL-6 was detected with mouse anti-biotin antibody (1:1.000, Abcam, Cambridge, United Kingdom), followed by anti-mouse Alexa Fluor 488 secondary antibody (1:1.000, Cell Signaling Technology). The cells were analysed on FACSCalibur flow cytometer (Becton Dickinson, Franklin Lakes, NJ, USA) by measuring the geometric mean fluorescence intensity (MFI) of at least 20.000 bacterial cells at 488 nm excitation and 530 nm emission wavelength. The data were analyzed with the FlowJo V10 software.

### Confocal fluorescence microscopy

Immunostaining of bacteria for fluorescence microscopy was carried out essentially as described above for flow cytometry, with the exception that the Alexa 555-conjugated goat anti-mouse antibody (1:1.000, Cell Signaling) was used to detect anti-flag antibodies. Stained cells were fixed on microscope slides coated with poly-L-lysine (Sigma-Aldrich) using a cytocentrifuge StatSpin Cytofuge 2 (Beckman Coulter, Brea, CA, USA). The prepared samples were examined with LSM 710 confocal microscope (Carl Zeiss, Oberkochen, Germany). The images were analyzed and processed with Image J version 1.52a [59].

### Cytokine quantification by ELISA

Quantification of cytokine binding by the engineered *L. lactis* was determined by enzyme-linked immunosorbent assay (ELISA) as previously described [51]. Commercially available human IL-6, TNF, IL-17, IL-23, IL-8, or mouse IL-6 ELISA kits were used (all from Mabtech, Nacka Strand, Sweden). The assay was carried out essentially according to manufacturer instructions. Briefly, cytokine-specific capture antibodies were immobilized on the surface of Nunc Maxisorp 96-well plates with high protein binding capacity (Thermo Fisher Scientific). After washing with PBS, wells were blocked with PBS containing 0.05% Tween-20 and 0.1% bovine serum albumin for 1 h at room temperature. All further washing steps were performed with PBS containing 0.05% Tween-20 (PBST). Recombinant cytokine standards from the ELISA kits (*Escherichia coli*-expressed, Mabtech) were spiked to incubation buffer (PBST with 0.1% bovine serum albumin) at 300 pg/mL (for IL-6: at 150, 300, 600, and 1200 pg/mL) and exposed to different concentrations of bacterial cells (eight twofold dilutions from 3 × 10^6^ to 6 × 10^9^ CFU/mL) for 2 h at room temperature with shaking. Bacterial cells were then pelleted and 200 µL aliquots of solution were loaded into the coated wells and incubated for 2 h at room temperature. For detection, biotin-conjugated cytokine-specific monoclonal antibodies were added to the wells (at recommended dilution) and incubated for 1 h at room temperature, followed by streptavidin–horseradish peroxidase (diluted 1:1000). The reaction was developed with 3,3’,5,5’-tetramethylbenzidine substrate (Sigma-Aldrich) and terminated after 10–30 min with 2 M sulfuric acid. Absorbances were measured at 450 nm on a Tecan Infinite M1000 (Salzburg, Austria). Cytokine levels were determined from calibration curves generated with different concentrations of recombinant cytokine standards from the ELISA kits (Mabtech). To ensure a uniform matrix effect, samples were incubated under the same conditions and in the same buffers as the cytokine standards. Binding was expressed as a percentage (%) of the cytokine that was removed from the solution by the bacteria. The concentration of bacterial cells necessary to remove 50% of IL-6 from the surrounding medium (half-maximal effective concentration, EC_50_) was determined by curve fitting with a four-parameter logistic regression model (4 PL) using Graph-Pad Prism 9.00 (San Diego, CA, USA).

### Cell culturing, differentiation and stimulation of cytokine production

The human monocytic leukemia cell line, THP-1 (TIB-202; American Type Culture Collection [ATCC], Manassas, Virginia, USA) and histiocytic lymphoma cell line, U-937 (CRL-1593.2; ATCC) were cultured in RPMI 1640 medium (Lonza, Basel, Switzerland) supplemented with 10% fetal bovine serum (Gibco, Carlsbad, CA, USA) and 1% penicillin/streptomycin (Gibco) at 37 °C and 5% CO_2_. Cell differentiation and stimulation were performed as described previously [51]. Briefly, the cells were seeded at 6 × 10^5^ cells/mL in 24-well plates (Corning, NY, USA), incubated overnight and then differentiated with 50 nM phorbol 12-myristate 13-acetate (PMA, Sigma-Aldrich). After 48 h incubation, the cells were allowed to recover for additional 48 h in a fresh complete RPMI medium without PMA. Differentiation was verified by observing changes in cell morphology under an Axio Observer Z1 epifluorescence microscope (Carl Zeiss). To induce the production of IL-6, the cells were treated with 1 µg/mL lipopolysaccharide (LPS) (L6529; Sigma-Aldrich) for 24 h. The supernatants of stimulated cells were collected and centrifuged (5 min, 2000 × *g* at 4 °C and 15 min, 1000 × *g* at 4 °C). IL-6 levels in the cell supernatants were determined as described above. If the values of produced IL-6 were above the linear range of the standard curve (10–1200 pg/mL), the supernatants were diluted. For cytokine removal experiments, the supernatants of the stimulated cells were incubated with the engineered bacteria for 2 h at room temperature with shaking. The amounts of the remaining IL-6 in the cell culture supernatants were measured as described above.

### Statistical analysis

Statistical analysis was performed using Graph-Pad Prism 9.00. Significant differences were determined using the unpaired Student’s t-test. Data were considered significant when P values were less than 0.05.

## Supplementary Information

**Additional file 1: Fig. S1.** Representative image of Coomassie blue-stained SDS-PAGE gel (left) and western blot (right) showing expression of IL-6-binding affibody ZIL in the whole cell lysate of induced and uninduced L. lactis bacterial cultures. ZIL, L. lactis harboring plasmid pSD-ZIL. ZIL-flag, L. lactis harboring plasmid pSD-ZIL-flag. Cont., L. lactis containing empty plasmid pNZ8148. Arrows are pointing to ZIL and ZIL-flag fusion proteins. **Fig. S2.** ELISA assay confirms that IL-6 binds to ZIL moiety of the fusion protein displayed on L. lactis surface and not to its other components (Usp-flag or AcmA). L. lactis displaying Usp-flag and AcmA in combination with nonrelevant binders IL-8-binding evasin (EVA) or HER2-binding affibody (ZHER) were used as negative controls. The experiment was performed in triplicate. Data are means ± standard deviation (SD). **Fig. S3.** THP-1 and U-937 cells differentiate into macrophage-like cells after exposure to phorbol 12-myristate 13-acetate (PMA) and secrete high amounts of IL-6 upon lipopolysaccharide (LPS) treatment. (a) Representative phase contrast microscopy images of untreated and PMA-treated THP-1 cells and U937 cells at 100X magnification. The cells (6 × 105 cells/mL) were incubated for 48 h in the absence or presence of PMA (50 nM), followed by 48 h recovery period in complete medium. The arrows indicate morphological changes after PMA treatment. (b) Time-course IL-6 secretion from differentiated THP-1 cells and differentiated U-937 cells induced with LPS (1 μg/mL). The culture supernatant was assayed for IL-6 at different time points by enzyme-linked immunosorbent assay (ELISA). Data are means ± standard deviation (SD) of three individual measurements.

## Data Availability

All data generated or analysed during this study are included in this published article.

## Abbreviations

CFU: Colony forming unit
EC_50_: Half-maximal effective concentration
ELISA: Enzyme-linked immunosorbent assay
GIT: Gastrointestinal tract
IBD: Inflammatory bowel disease
IL: Interleukin
LAB: Lactic acid bacteria
MFI: Mean fluorescence intensity
MCS: Multiple cloning site
PBS: Phosphate-buffered saline
PMA: Phorbol 12-myristate 13-acetate
TBS: Tris-buffered saline
TNF: Tumor necrosis factor
