# Supplementary material for: Targeting IL-6 by engineered Lactococcus lactis via surface-displayed affibody
Source: Microb Cell Fact. 2022 Jul 16;21:143. doi: 10.1186/s12934-022-01873-7 (PMC9287920; doi:10.1186/s12934-022-01873-7)
Supplement: Supplementary file 1 — Additional file 1: Fig. S1. Representative image of Coomassie blue-stained SDS-PAGE gel (left) and western blot (right) showing expression of IL-6-binding affibody ZIL in the whole cell lysate of induced and uninduced L. lactis bacterial cultures. ZIL, L. lactis harboring plasmid pSD-ZIL. ZIL-flag, L. lactis harboring plasmid pSD-ZIL-flag. Cont., L. lactis containing empty plasmid pNZ8148. Arrows are pointing to ZIL and ZIL-flag fusion proteins. Fig. S2. ELISA assay confirms that IL-6 binds to ZIL moiety of the fusion protein displayed on L. lactis surface and not to its other components (Usp-flag or AcmA). L. lactis displaying Usp-flag and AcmA in combination with nonrelevant binders IL-8-binding evasin (EVA) or HER2-binding affibody (ZHER) were used as negative controls. The experiment was performed in triplicate. Data are means ± standard deviation (SD). Fig. S3. THP-1 and U-937 cells differentiate into macrophage-like cells after exposure to phorbol 12-myristate 13-acetate (PMA) and secrete high amounts of IL-6 upon lipopolysaccharide (LPS) treatment. (a) Representative phase contrast microscopy images of untreated and PMA-treated THP-1 cells and U937 cells at 100X magnification. The cells (6 × 10^5^ cells/mL) were incubated for 48 h in the absence or presence of PMA (50 nM), followed by 48 h recovery period in complete medium. The arrows indicate morphological changes after PMA treatment. (b) Time-course IL-6 secretion from differentiated THP-1 cells and differentiated U-937 cells induced with LPS (1 μg/mL). The culture supernatant was assayed for IL-6 at different time points by enzyme-linked immunosorbent assay (ELISA). Data are means ± standard deviation (SD) of three individual measurements. [file 12934_2022_1873_MOESM1_ESM.pdf]

# Additional Material

## Targeting IL-6 by engineered *Lactococcus lactis* via surface-displayed affibody

**Abida Zahirović[1], Aleš Berlec[1,2*]**

[1]Department of Biotechnology, Jožef Stefan Institute, Ljubljana, Slovenia

[2]Faculty of Pharmacy, University of Ljubljana, Ljubljana, Slovenia

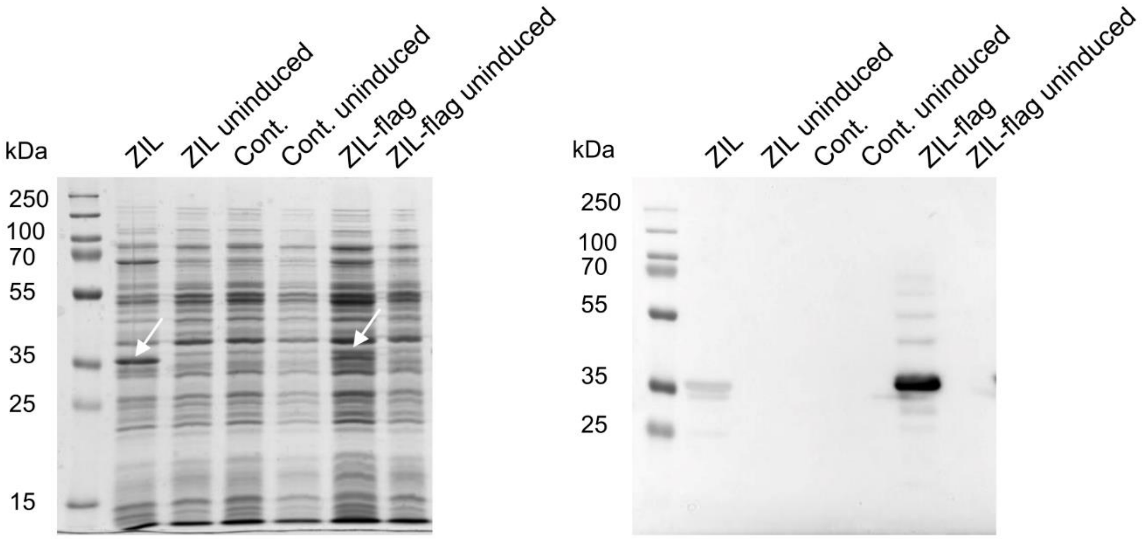


**Additional file 1: Fig. S1 Representative image of Coomassie blue-stained SDS-PAGE gel (left) and western blot (right) showing expression of IL-6-binding affibody ZIL in the whole cell lysate of induced and uninduced *L. lactis* bacterial cultures.** ZIL, *L. lactis* harboring plasmid pSD-ZIL. ZIL-flag, *L. lactis* harboring plasmid pSD-ZIL-flag. Cont., *L. lactis* containing empty plasmid pNZ8148. Arrows are pointing to ZIL and ZIL-flag fusion proteins.

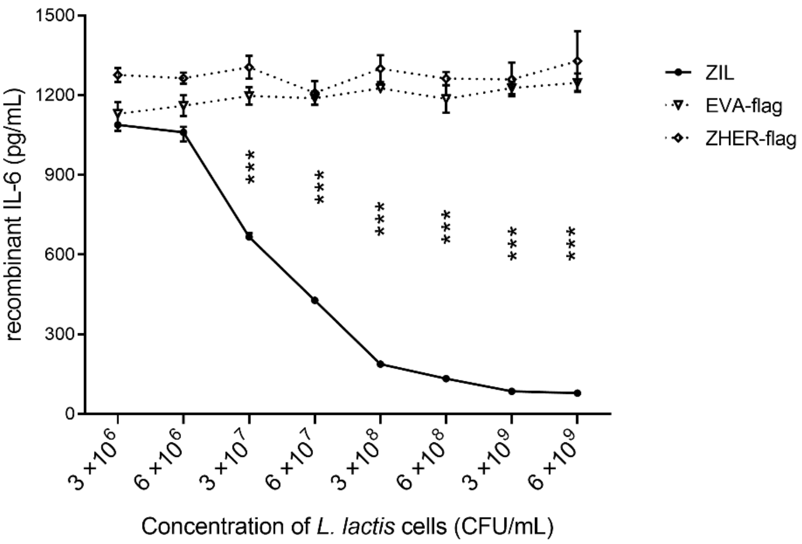


**Additional file 1: Fig. S2 ELISA assay confirms that IL-6 binds to ZIL moiety of the fusion protein displayed on L. lactis surface and not to its other components (Usp-flag or AcmA).** L. lactis displaying Usp-flag and AcmA in combination with nonrelevant binders IL-8-binding evasin (EVA) or HER2-binding affibody (ZHER) were used as negative controls. The experiment was performed in triplicate. Data are means ±standard deviation (SD).

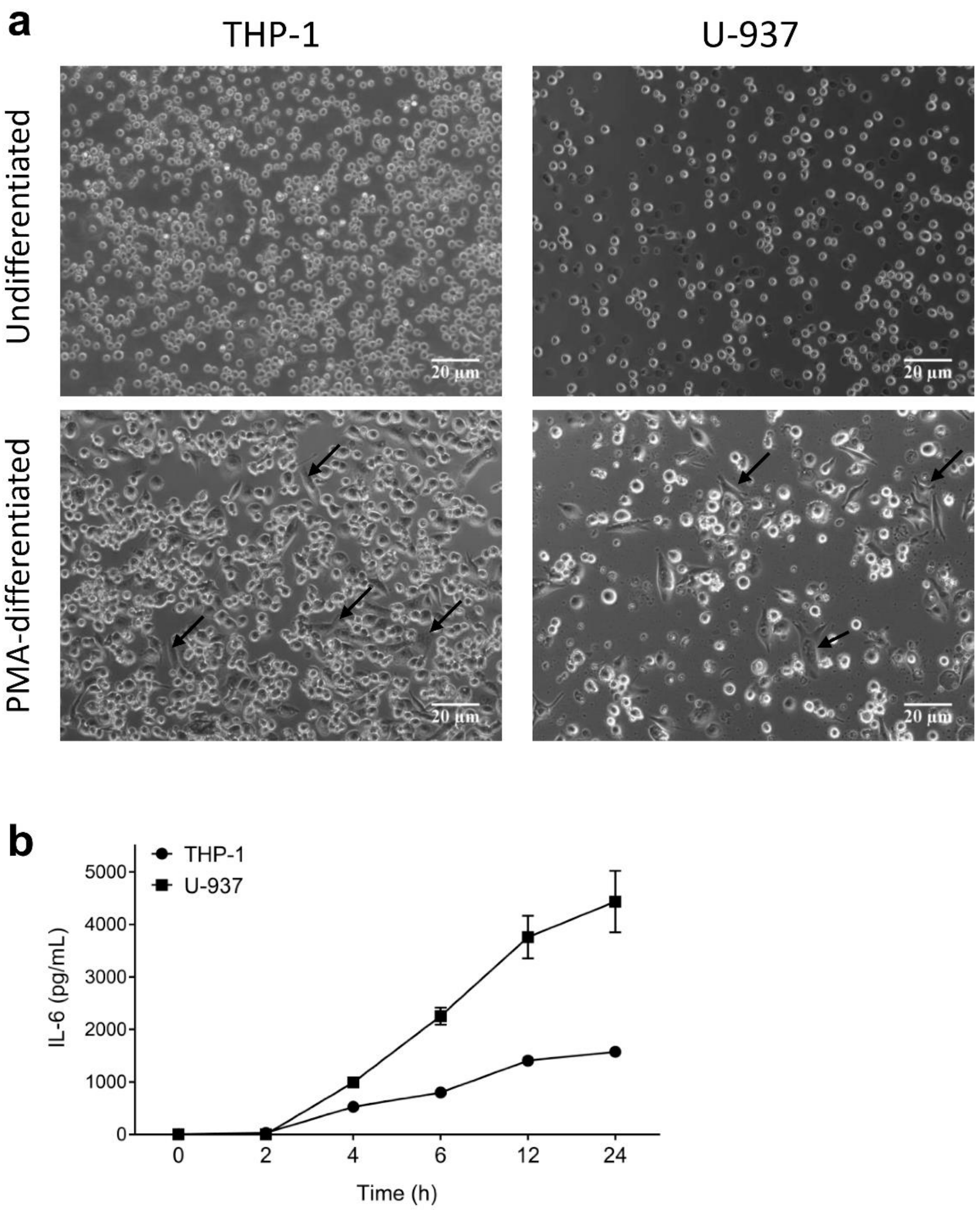


**Additional file 1: Fig. S3 THP-1 and U-937 cells differentiate into macrophage-like cells after exposure to phorbol 12-myristate 13-acetate (PMA) and secrete high amounts of IL-6 upon lipopolysaccharide (LPS) treatment.** (**a**) Representative phase contrast microscopy images of untreated and PMA-treated THP-1 cells and U937 cells at 100X magnification. The cells ($6 \times 10^5$ cells/mL) were incubated for 48 h in the absence or presence of PMA (50 nM), followed by 48 h recovery period in complete medium. The arrows indicate morphological changes after PMA treatment. (**b**) Time-course IL-6 secretion from differentiated THP-1 cells and differentiated U-937 cells induced with LPS (1 µg/mL). The culture supernatant was assayed for IL-6 at different time points by enzyme-linked immunosorbent assay (ELISA). Data are means ±standard deviation (SD) of three individual measurements.
